# Predicting mortality in patients undergoing VA-ECMO after coronary artery bypass grafting: the REMEMBER score

**DOI:** 10.1186/s13054-019-2307-y

**Published:** 2019-01-11

**Authors:** Liangshan Wang, Feng Yang, Xiaomeng Wang, Haixiu Xie, Eddy Fan, Mark Ogino, Daniel Brodie, Hong Wang, Xiaotong Hou

**Affiliations:** 1 Center for Cardiac Intensive Care, Beijing Anzhen Hospital, Capital Medical University, Beijing, People’s Republic of China; 2 Interdepartmental Division of Critical Care Medicine, University of Toronto, Toronto, Ontario Canada; 3 Division of Neonatology, Nemours/Alfred I. DuPont Hospital for Children, Wilmington, Delaware, USA; 4 Columbia University College of Physicians and Surgeons/New York-Presbyterian Hospital, New York, NY USA

**Keywords:** Cardiogenic shock, Venoarterial extracorporeal membrane oxygenation, Coronary artery bypass grafting, pRedicting mortality in patients undergoing veno-arterial Extracorporeal MEMBrane oxygenation after coronary artEry bypass gRafting (REMEMBER) score, Mortality

## Abstract

**Background:**

Prediction scoring systems for coronary artery bypass grafting (CABG) patients on venoarterial extracorporeal membrane oxygenation (VA-ECMO) have not yet been reported. This study was designed to develop a predictive score for in-hospital mortality for cardiogenic shock patients who received VA-ECMO after isolated CABG.

**Methods:**

Retrospective cohort study of consecutive CABG patients supported with VA-ECMO (*n* = 166) at the Beijing Anzhen Hospital between February 2004 and March 2017.

**Results:**

One hundred and six patients (64%) could be weaned from VA-ECMO, and 74 patients (45%) survived to hospital discharge. On the basis of multivariable logistic regression analyses, the pRedicting mortality in patients undergoing veno-arterial Extracorporeal MEMBrane oxygenation after coronary artEry bypass gRafting (REMEMBER) score was created with six pre-ECMO parameters: older age, left main coronary artery disease, inotropic score > 75, CK-MB > 130 IU/L, serum creatinine > 150 umol/L, and platelet count < 100 × 10^9^/L. Four risk classes, namely class I (REMEMBER score 0–13), class II (14–19), class III (20–25), and class IV (> 25) with their corresponding mortality (13%, 55%, 70%, and 94%, respectively), were identified. The area under the receiver operating characteristic curve 0.85(95% CI 0.79–0.91) for the REMEMBER score was better than those for the SOFA, SAVE, EuroSCORE, and ENCOURAGE scores in this population.

**Conclusions:**

The REMEMBER score might help clinicians at bedside to predict in-hospital mortality for patients receiving VA-ECMO after isolated CABG for refractory cardiogenic shock. Prospective studies are needed to externally validate this scoring system.

**Electronic supplementary material:**

The online version of this article (10.1186/s13054-019-2307-y) contains supplementary material, which is available to authorized users.

## Background

Approximately 1% of patients who undergo coronary artery bypass grafting (CABG) experience refractory postcardiotomy cardiogenic shock (PCS), which is associated with increased morbidity and mortality [1–3]. In these patients, venoarterial extracorporeal membrane oxygenation (VA-ECMO) may be considered as a rescue strategy to provide temporary circulatory and respiratory support allowing cardiac function recovery or bridging to additional therapeutic alternatives [4–6]. Despite major innovations in ECMO support over the last few decades, in-hospital mortality from cardiogenic shock in post-CABG patients supported with VA-ECMO remains high [1–3, 5–13]. Early identification of pre-ECMO factors associated with mortality may prognosticate in post-CABG patients. In this context, the survival after VA-ECMO (SAVE) score for refractory cardiogenic shock in general and the prEdictioN of Cardiogenic shock OUtcome foR Acute myocardial infarction patients salvaGed by VA-ECMO (ENCOURAGE) risk score have been published over the past few years [14, 15]. However, there are currently few studies reporting mortality risk factors and clinical outcomes of PCS patients who were supported with VA-ECMO after CABG [6, 7]. In addition, prediction scoring systems for CABG patients on VA-ECMO have not yet been reported. We developed the pRedicting mortality in patients undergoing veno-arterial Extracorporeal MEMBrane oxygenation after coronary artEry bypass gRafting (REMEMBER) score, which might help the clinicians to select patients that would benefit from VA-ECMO after CABG.

## Materials and methods

### Patients

We retrospectively evaluated consecutive patients who received VA-ECMO between February 2004 and March 2017 at the Beijing Anzhen Hospital. Patients were enrolled in the study if they received VA-ECMO treatment for refractory PCS after isolated CABG. The clinical criteria for PCS [2, 16] included the following: left atrial pressure > 15 mmHg; central venous pressure > 12 mmHg; metabolic acidosis (i.e., pH < 7.3 with serum lactate > 3.0 mmol/L); end-organ hypoperfusion (urine output < 30 mL/h); cardiac index < 2.2 L/min/m^2^; and systolic blood pressure < 80 mmHg despite adequate filling volumes, use of multiple adrenergic agents (epinephrine > 0.1 μg/kg/min or dobutamine > 10 μg/kg/min, norepinephrine > 0.1 μg/kg/min), or an intra-aortic balloon pump (IABP). Exclusion criteria for patient selection from our institutional ECMO database were an age < 18 years, venovenous ECMO support for acute respiratory failure, ECMO initiation before CABG or at more than 7 days after CABG, and concomitant other major cardiac procedures (valvular replacement, valvuloplasty, or aortic surgery). The study was approved by the institutional ethics committee/review board of the Beijing Anzhen Hospital (2016005X), and the requirement for informed patient consent was waived in view of the retrospective nature of the study.

### ECMO implantation and management

The details regarding VA-ECMO initiation and management have been described previously [3]. Briefly, VA-ECMO support was initiated via peripheral cannulation through the femoral route with the semi-open method, and an additional 6 Fr catheter was systematically inserted distally into the femoral artery to prevent severe leg ischemia. All procedures were performed by trained ECMO team members. ECMO blood flow was adjusted based on clinical assessments (e.g., mixed venous oxygen saturation, evidence of hypoperfusion, resolution of hyperlactatemia, normalization of mean arterial pressure). Intravenous unfractionated heparin was given to maintain an activated clotting time of 180–210 s, or an activated partial thromboplastin time of 1.5–2 times normal. ECMO-related complications were carefully monitored. When patients fulfilled our institutional weaning criteria, a protocolized weaning trial was performed [17, 18]. Weaning was considered unsuccessful if ECMO re-cannulation was required within 2 days of decannulation. (See supplementary material online for weaning criteria and data collection.)

### Outcomes and definitions

The primary outcome was in-hospital mortality, defined as death from any cause occurring in patients who were treated by VA-ECMO for PCS post CABG. Secondary outcomes included ECMO duration, length of intensive care unit (ICU) stay, length of hospital stay, survival to ECMO weaning, continuous renal replacement therapy (CRRT), systemic infection, bleeding requiring thoracotomy, major neurological complications, and major ECMO-related complications. Systemic infection was defined by a positive blood culture. Major neurological complications included brain death, ischemic stroke, hemorrhagic stroke, and anoxic encephalopathy. Major ECMO-related complications included femoral hemorrhage due to arterial laceration, leg ischemia requiring surgical intervention (fasciotomy or amputation), infection at the site of ECMO cannula insertion, cannula thrombosis, and need for an oxygenator change.

### Statistical analysis

All the analyses were performed with STATA /SE 12.0 (StataCorp, College Station, TX, USA) and SPSS 19.0 (SPSS Inc., Chicago, IL, USA). The characteristics of patients were reported as proportions for categorical variables and as median (interquartile range (IQR)) for continuous variables. Categorical variables were compared with chi-square or Fisher’s exact tests, and continuous variables were compared with Student’s *t* test or the Mann–Whitney *U* test, as appropriate. The cumulative rates of survival after ECMO initiation were analyzed using the Kaplan–Meier method, and inter-group comparisons were performed using the log-rank test. *P* values less than 0.05 were considered to be statistically significant.

The REMEMBER (pRedicting mortality in patients undergoing veno-arterial Extracorporeal MEMBrane oxygenation after coronary artEry bypass gRafting) score was developed according to published recommendations and using multivariable regression analysis [19, 20]. Briefly, the following steps was used.

- Step 1: Identification of “candidate predictors”

Patients’ demographic, clinical, and biological characteristics prior to ECMO initiation were considered. All candidate predictors of mortality were assessed with univariable logistic regression. Continuous variables were converted into categorical variables for practical purposes. Variables associated with mortality (*p* ≤ 0.2) were included in the multivariable model. The following known prognostic factors were forced into the final multivariable model irrespective of their statistical significance: pulmonary disease, pre-ECMO cardiac arrest, and ECMO initiation during cardiopulmonary resuscitation (CPR). All potential variables included in the multivariable analyses were subjected to a correlation matrix for analysis of multicollinearity.

- Step 2: Construction of the REMEMBER score

The multivariate logistic regression model was built using a backward stepwise selection process in which variables were removed from the model at each step based on the *p* value of more than 0.1. Only variables with *p* values ≤ 0.05 were retained in the final multivariable model. Regression *β* coefficients and their 95% confidence intervals (CIs) were re-estimated by logistic regression with bootstrapping, sampling the whole data using 1000 repetitions with replacement [21]. To derive practical REMEMBER component scores, each factor’s *β* coefficient retained in the logistic regression model was divided by the model’s smallest coefficient, multiplied by 5, and rounded to the nearest integer.

- Step 3: Internal validation

Logistic regression was used to reassess score performance in the original dataset. Model discrimination and calibration were assessed using the area under the receiver operating characteristics curve (AUROC) and the Hosmer-Lemeshow *C*-statistic, respectively. Model discrimination of the REMEMBER score versus the Sepsis-related Organ Failure Assessment (SOFA) score [22], SAVE score [14], ENCOURAGE score [15], and EuroSCORE [23] were compared using AUROC. Moreover, a sensitivity analysis was performed to determine the performance of the REMEMBER score in over 2 time periods: early (2004–2012) versus late (2013–2017).

## Results

### Populations

Five hundred and seventy-seven patients underwent 580 ECMO runs over the 14-year period. Among those patients, 315 patients who did not undergo CABG were excluded. Finally, 166 patients were retained as the derivation cohort to create the REMEMBER score (Additional file 1: Figure S1). Their demographic and pre-ECMO characteristics are presented in Table 1 and Additional file 1: Table S3. Briefly, most of the patients (78%) were diagnosed with unstable angina, and 52 patients (31%) had left main coronary artery disease. Eighty-three patients (50%) underwent off-pump CABG. Sixty patients (36%) were not successfully weaned from cardiopulmonary bypass (CPB) due to PCS requiring transition to ECMO. Ninety-one patients (55%) suffered from cardiac arrest before VA-ECMO implantation. One hundred and thirty-two patients (80%) had an IABP placed before ECMO insertion. Sixty-two percent of all runs occurred in the late period from 2013 to 2017 (Additional file 1: Figure S2 and Table S3).

**Table 1:** Clinical characteristics of the patients at ECMO initiation according to hospital survival status

Characteristic	All patients (*n* = 166)	Survivors (*n* = 74)	Non-survivors (*n* = 92)	*p* value
Age, years	61 (54–67)	57 (50–64)	63 (58–68)	< 0.001
Male	132 (80)	64 (86)	68 (74)	0.046
Weight, kg	70 (63–80)	73 (64–80)	70 (63–80)	0.173
Comorbid conditions				
Hypertension	99 (60)	38 (51)	61 (66)	0.051
Diabetes	58 (35)	19 (26)	39 (23)	0.025
CCS class 4 angina	55 (33)	20 (27)	35 (38)	0.134
Smoking	92 (55)	47 (64)	45 (49)	0.060
Chronic pulmonary disease	5 (3)	3 (4)	2 (2)	0.481
Left main disease^a^	52 (31)	13 (18)	39 (42)	0.001
Diagnosis				
Unstable angina	129 (78)	58 (78)	71 (77)	0.853
NSTMI	11 (7)	3 (4)	8 (9)	0.378
STMI	26 (16)	13 (18)	13 (14)	0.545
EuroSCORE	6 (4–7)	5 (3–6)	6 (5–8)	< 0.001
Operative parameters				
Emergency operation	25 (15)	8 (11)	17 (18)	0.170
OPCABG	83 (50)	38 (51)	45 (49)	0.755
Conversion to on-pump	44 (27)	19 (26)	25 (27)	0.828
LIMA graft	120 (72)	55 (74)	65 (71)	0.599
Number of distal anastomoses	3 (2–4)	3 (2–4)	3 (3–3)	0.648
Unsuccessful weaning off CPB	60 (36)	28 (38)	32 (35)	0.684
Pre-ECMO cardiac arrest	91 (55)	40 (54)	51 (55)	0.859
ECMO initiation during CPR	28 (17)	11 (15)	17 (18)	0.537
Right ventricular failure	11 (7)	4 (5)	7 (8)	0.571
Pre-CABG IABP	32 (13)	10 (14)	22 (24)	0.091
Pre-ECMO IABP	132 (80)	62 (84)	70 (75)	0.222
IABP insertion during ECMO	7 (4)	1 (1)	6 (7)	0.208
SOFA score	12 (10–13)	11 (9–13)	13 (12–14)	< 0.001
Inotropic score*^b^	75 (55–93)	58 (43–83)	82 (67–99)	< 0.001
Epinephrine^c^, μg/kg/min	0.5 (0.35–0.69)	0.40 (0.28–0.65)	0.57 (0.40–0.70)	< 0.001
Dobutamine^d^, μg/kg/min	12 (8–16)	10 (8–15)	15 (10–19)	< 0.001
Norepinephrine^e^, μg/kg/min	0.1 (0.06–0.18)	0.1 (0.05–0.15)	0.1 (0.06–0.2)	0.062
Pre-ECMO blood pressure^b^				
SAP, mmHg	70 (58–78)	70 (59–79)	70 (55–77)	0.454
DAP, mmHg	40 (30–50)	40 (32–50)	40 (30–45)	0.163
MAP, mmHg	55 (45–63)	55 (49–65)	55 (45–61)	0.285
Biological parameters^b^				
PH	7.35 (7.30–7.39)	7.37 (7.32–7.41)	7.34 (7.28–7.39)	0.058
Serum lactate, mmol/L	11.4 (7.8–17.4)	9.0 (5.5–14.5)	13.8 (9.4–18.4)	< 0.001
Hemoglobin, g/dL	9.8 (8.6–10.7)	10.1 (9.2–10.8)	9.2 (8.3–10.6)	0.033
Platelet, ×10^9^/L	103 (58–153)	117 (63–165)	86 (52–132)	0.111
Serum Creatinine^f^, umol/L,	121 (91–160)	96 (80–130)	141 (108–197)	< 0.001
CK-MB^g^, IU/L	143 (48–259)	103 (46–243)	157 (59–281)	0.040

Data are presented as medians (25th–75th percentile) or *n* (%)

*CCS*, Canadian Cardiovascular Society classification of angina; *NSTMI*, non ST-elevation myocardial infarction; *STMI*, ST-elevation myocardial infarction; *CABG*, coronary artery bypass grafting; *IABP*, intra-aortic balloon pump; *OPCABG*, off-pump CABG; *LIMA*, left internal mammary artery; *CPB*, cardiopulmonary bypass; *ECMO*, extracorporeal membrane oxygenation; *CPR*, cardiopulmonary resuscitation; *SOFA score*, the Sequential Organ Failure Assessment score; *SAP*, *DAP*, and *MAP*, systolic, diastolic, and mean arterial blood pressure, respectively; *CK-MB*, creatine kinase-MB

*Inotropic score, in μg/kg/min, was calculated as follows: dopamine + dobutamine + 100 × epinephrine + 100 × norepinephrine + 15 × milrinone

^a^Left main disease was defined as any stenosis ≥ 50% of the left main trunk

^b^Worse value within 6 h prior ECMO cannulation

^c^All of the patients were treated with epinephrine

^d^One hundred and sixty-three patients were treated with dobutamine (73 survivors and 90 non-survivors)

^e^One hundred and thirty-nine patients were treated with norepinephrine (62 survivors and 77 non-survivors)

^f^Values were obtained for 157/166 patients (69 survivors and 88 non-survivors)

^g^Values were obtained for 160/166 patients (73 survivors and 87 non-survivors)

### Patient outcomes

Ninety-two patients (55%) died in hospital (Additional file 1: Table S1). Eighty-two deaths (49%) were attributed to multi-organ failure, 7 (4%) were with anoxic encephalopathy or brain death, and 3 (2%) patients died of cardiac arrest (Additional file 1: Figure S3). One hundred and six patients (64%) could be weaned from VA-ECMO. The median (IQR) time on VA-ECMO support was 4 (3–6) days. More than 60% of the patients used ECMO for 3–6 days, and these patients had significantly lower mortality than those who used ECMO for < 3 days (41% vs 81%, *p* < 0.001) or ≥ 7 days (41% vs 76%, *p* = 0.002) (Additional file 1: Figure S4). The median (IQR) length of ICU stay and hospital stay duration were 8 (5–12) and 20 (13–30) days, respectively. Twenty-five (15%) patients underwent repeat thoracotomy for bleeding. Major neurological complications were found in 26 (16%) of the patients. Major ECMO-related complications occurred in 57 (34%) of the patients.

### Predictors of in-hospital mortality

Continuous variables were converted into categorical variables (Additional file 1: Table S4). Variables associated with mortality at the time of ECMO initiation by univariable analysis were older age, female, weight ≥ 83 kg, hypertension, diabetes, Canadian Cardiovascular Society (CCS) class 4 angina, smoking, left main coronary artery disease, pre-CABG IABP, emergency operation, inotropic score [24] > 75, diastolic pressure ≤ 45 mmHg, pre-ECMO lactate > 9 mmol/L, hemoglobin < 10 g/dL, platelet count < 100 × 10^9^/L, serum creatinine > 150 umol/L, and creatine kinase-MB (CK-MB) > 130 IU/L (Additional file 1: Table S2). Multivariable logistic regression analysis identified older age, left main coronary artery disease, inotropic score > 75, CK-MB > 130 IU/L, serum creatinine > 150 umol/L, and platelet count < 100 × 10^9^/L as independent risk factors associated with in-hospital mortality (Table 2). Bootstrap analysis showed the similar results, confirming the stability of the original model (Additional file 1: Table S5).

**Table 2:** Results of multivariable analyses and the REMEMBER score

Parameter	*β* Coefficient	OR (95% CI)	*p* value	Score
Age, years				
< 54	0	1		0
54–67	1.783	5.95 (2.08–17.06)	0.001	8
> 67	2.384	10.85 (2.71–43.41)	0.001	11
Left main disease^a^	1.625	5.08 (2.05–12.57)	< 0.001	7
Inotropic score >75^b^	1.126	3.08 (1.32–7.21)	0.009	5
CK-MB > 130 IU/L^b^	1.145	3.14 (1.36–7.24)	0.007	5
Serum creatinine > 150 umol/L^b^	1.496	4.46 (1.73–11.53)	0.002	7
Platelet count < 100 × 10^9^/L^b^	1.271	3.56 (1.50–8.50)	0.004	6

The prediction equation is:1/(1 + exp. (3.856–0.220*score)). Hosmer–Lemeshow χ^2^, 6.029 with 8 df; *p* = 0.644

*OR* odds ratio, *CK-MB* creatine kinase-MB

^a^Left main disease was defined as any stenosis ≥ 50% of the left main trunk

^b^Worse value within 6 h prior ECMO cannulation

### REMEMBER score

**Fig. 1:**
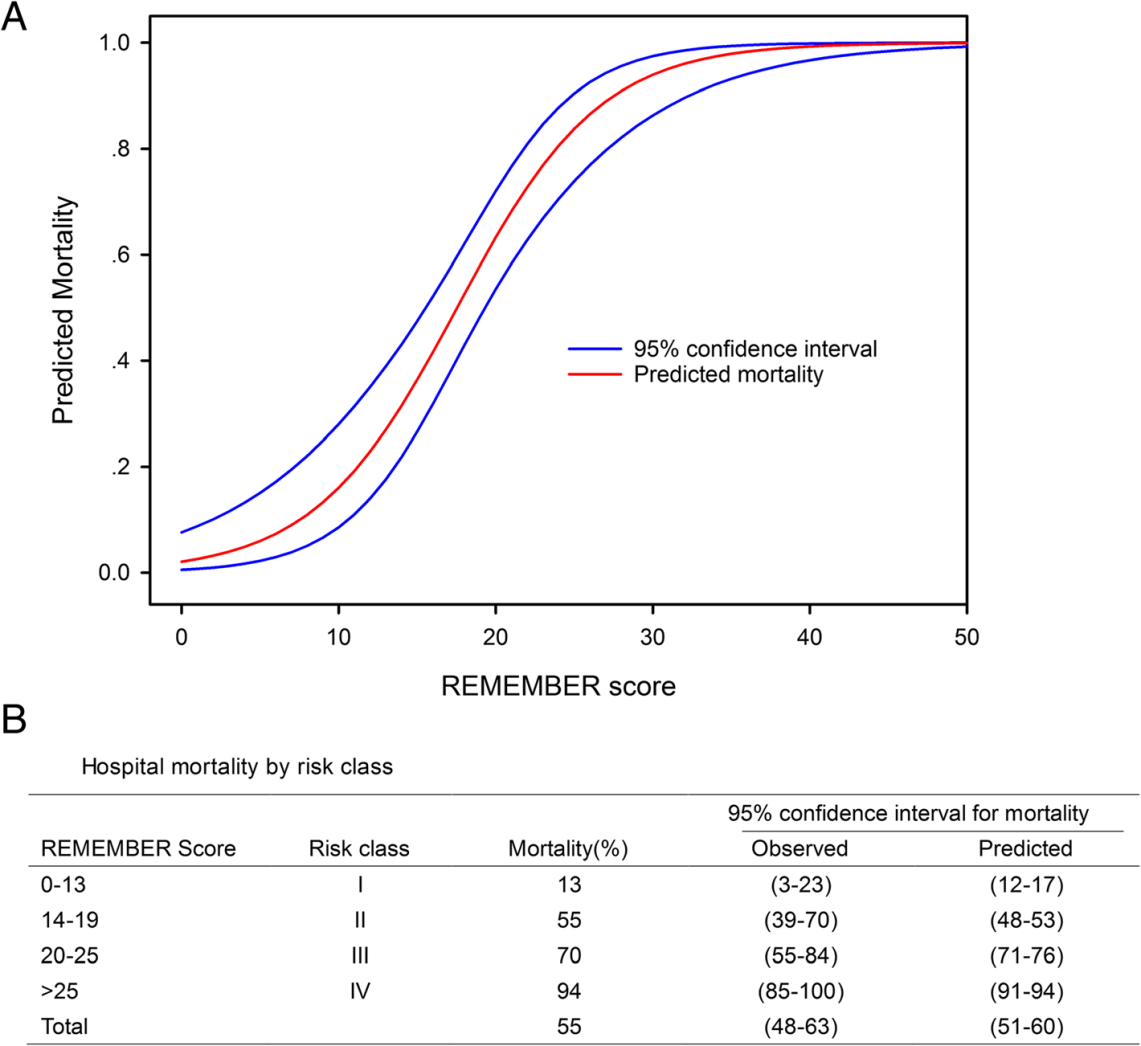
**a** Predicted mortality at each score level. Curved blue lines represent 95% confidence interval for predicted survival at each score level. **b** Hospital mortality by risk class. Observed mortality and predicted mortality are expressed as mean ± 95% confidence interval

**Fig. 2:**
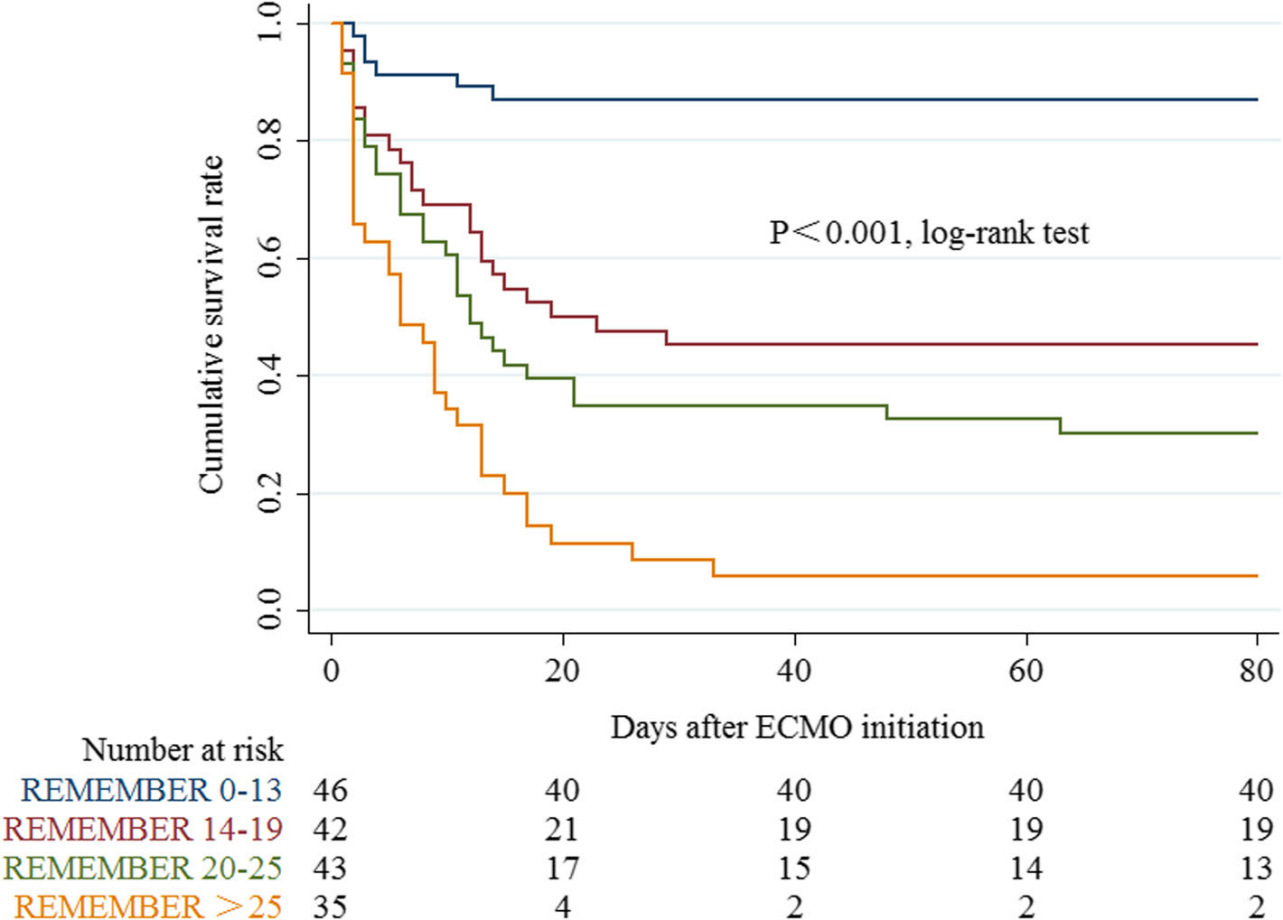
Kaplan–Meier estimates of cumulative probabilities of 80-day survival for patients with the indicated pre-ECMO REMEMBER-score classes

**Fig. 3:**
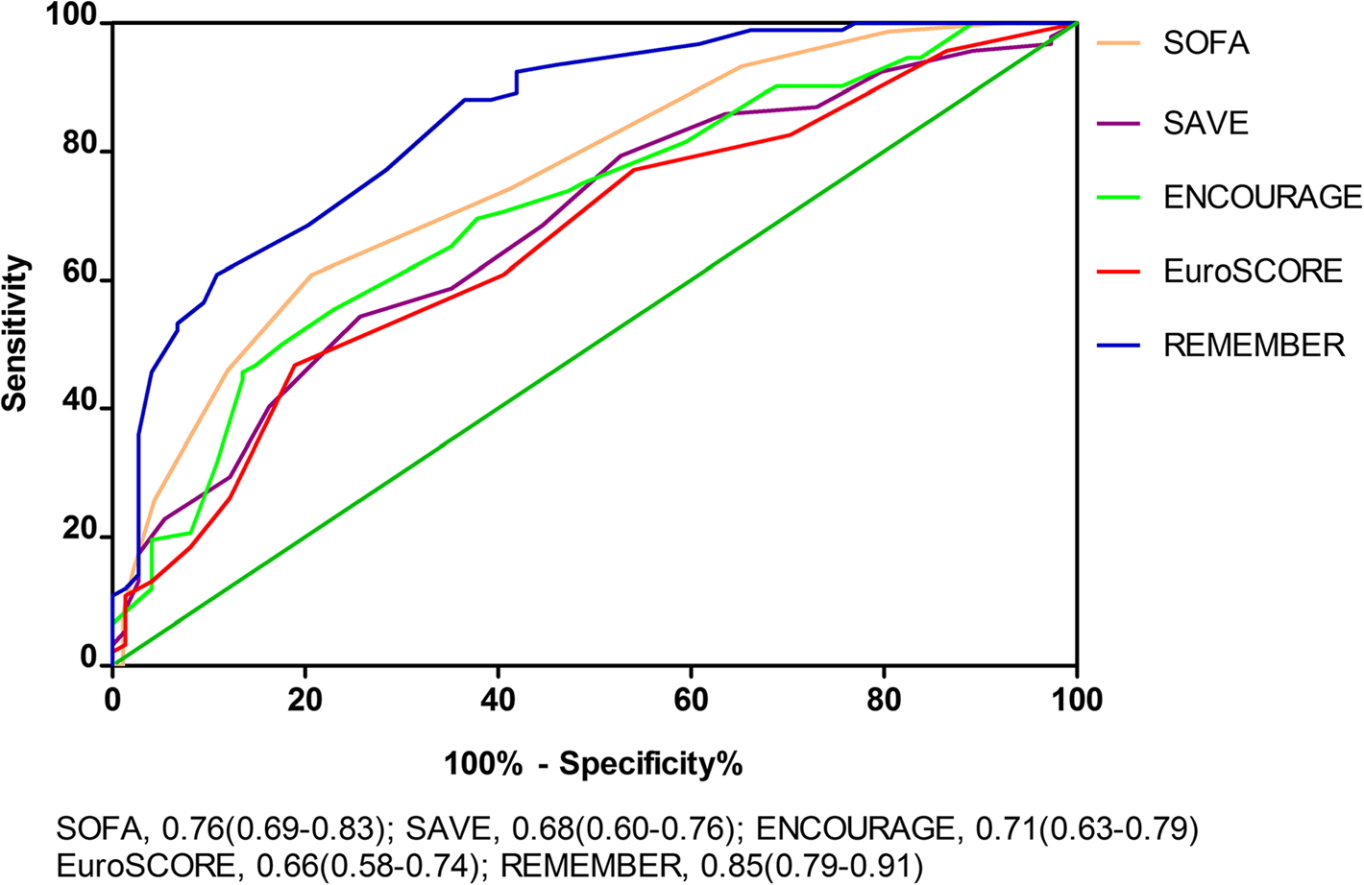
The areas under the receiver operating characteristic curves for predicting in-hospital death. Discriminatory performance of REMEMBER score was greater than other scores

Six items were retained to create the REMEMBER score (Table 2). The prediction equation is 1/(1 + exp. (3.856–0.220*score)). Individual predicted in-hospital CABG-ECMO mortality risk is calculated by applying the REMEMBER score to Fig. 1, which displays the 95% CI for mortality of the development dataset used to derive the score. REMEMBER score calibration was good (Hosmer–Lemeshow *χ*^2^ *p* = 0.644; Table 2). Four risk classes, namely class I (REMEMBER score 0–13), class II (14–19), class III (20–25), and class IV (> 25) with their corresponding mortality rates (13%, 55%, 70%, and 94%, respectively), were identified (Fig. 1 and Additional file 1: Figure S5). When the scoring system was applied in the four different risk groups, there was very good overlap between observed and expected mortality in all four groups (Fig. 1). Cumulative 80-day survival rate by risk class is shown in Fig. 2. The AUROC for the REMEMBER score was 0.85 (95% CI 0.79–0.91), which was better than those of the SOFA, SAVE, EuroSCORE and ENCOURAGE scores (Fig. 3). The REMEMBER score exhibited similar performance across both eras (2004–2012: *C* = 0.83 (95% CI 0.73–0.93); 2013–2017: *C* = 0.86 (95% CI 0.79–0.93); Additional file 1: Figure S6). Spearman rank correlation matrix for the different scoring systems is provided in Additional file 1: Table S6.

## Discussion

This single-center, retrospective study included 166 PCS patients supported with VA-ECMO after CABG over a 14-year period and showed that the in-hospital mortality rate was 55%. We identified risk factors associated with in-hospital death and developed a mortality risk score (REMEMBER score), which comprised 6 pre-ECMO variables, exhibited good performance, and is focused on patients with PCS following CABG, as compared to previous scores.

Although ECMO devices and intensive care management have undergone notable advances over the past two decades, short-term mortality associated with PCS remains unacceptably high at 45–75% [1–3, 5–13, 18, 25–28]. Older age, female sex, obesity, diabetes, renal insufficiency, pre-ECMO blood lactate, elevated CK-MB levels, low serum albumin level, low platelet count, poor cardiac systolic function, and logistic EuroSCORE more than 20% were independently associated with in-hospital death in PCS patients undergoing ECMO after cardiac-related procedures [1, 2, 7, 9, 11, 27–31]. It had been demonstrated that older age, female sex, diabetes, left main disease, and elevation of CK-MB levels were independent risk factors for death of CABG patients [32–36]. However, there is a paucity of data on patients receiving VA-ECMO after isolated CABG. In a recent retrospective study [7] involving 148 patients with PCS after CABG, in-hospital mortality was 64.2%, which was higher than that of our series. Moreover, the authors found that creatinine clearance, pulmonary disease, and pre-VA-ECMO blood lactate were independently associated with in-hospital death.

Despite these previously reported mortality risk factors, there are no published scoring system based on pre-ECMO parameters to predict the outcomes of VA-ECMO-supported CABG patients with PCS refractory to conventional treatment. The REMEMBER score incorporates six simple pre-ECMO variables and demonstrated good performance (AUROC 0.85) in the derivation cohort. All these parameters are readily measurable and available to clinicians before VA-ECMO implantation. Our study highlights the importance of older age in determining in-hospital death, which was reflected in its weighting in the REMEMBER score. The other five variables had similar weightings in the score. Our findings also confirmed that the presence of left main disease, elevated CK-MB, acute kidney injury, and thrombocytopenia at ECMO initiation were associated with poorer outcomes. In addition, inotropic score was related to short-term death, which was used to roughly estimate the severity of the pre-ECMO status [24].

Scoring systems are often used to select appropriate patients for specific therapies [37, 38]. However, for PCS patients who clinicians believe would die without VA-ECMO, decision-making by clinicians often involves many patient and contextual factors and remains difficult despite the use of prediction models. Risk stratification, however, will inform family members and clinicians of the likely risk of death for a group of patients with a similar risk profile undergoing the proposed operation. The present study identified four risk classes according to the REMEMBER score. The score might aid in family counseling and shared decision-making relative to clinical outcomes and help clinicians identify high-risk post-CABG patients who may suffer poor outcomes despite the use of the VA-ECMO.

In the past few years, the SAVE [14] and ENCOURAGE [15] scores have been developed to predict survival of patients receiving ECMO for refractory cardiogenic shock. The EuroSCORE [23] and SOFA [22] scores are widely used in the fields of cardiac surgery and critical care, respectively. Importantly, the REMEMBER score had better discrimination than these previously published scores in our cohort. In our scoring system, female patients were not at increased risk of death, which differed from that in the EuroSCORE [23] and ENCOURAGE [15] score. While female sex was significantly associated with mortality in univariable analyses, it was not retained in the final multivariable logistic regression model (*p* = 0.065). One possible explanation was the relatively small number of women in our study. Pre-ECMO cardiac arrest was found to be associated with the increased mortality in the SAVE score [14], whereas this phenomenon was not observed in our study. Most of these patients suffered from in-hospital cardiac arrest and had very short no- and low-flow times, which might account for our findings. Another difference between our score and the ENCOURAGE [15] score was our lack of an association between the serum lactate and in-hospital death, potentially owing to some patients surviving to hospital discharge despite initially high lactate levels.

Our study has several limitations. First, it was a single-center, retrospective study which may limit the generalizability of our results. Second, because left ventricular assist devices were not registered in China, no patients underwent ventricular assist device after VA-ECMO. The usefulness of VA-ECMO for CABG patients might have therefore been underestimated. Third, since patients requiring VA-ECMO before isolated CABG were not included in our study, the applicability of the REMEMBER score to these patients remains unknown. Fourth, half of the patients underwent off-pump CABG. On-pump CABG is the preferred surgical procedure for coronary artery disease patients in many other centers with extensive surgical experience [39, 40]. However, off-pump CABG was not associated with outcomes in the present study. Fifth, our institution tries to do VA-ECMO early which may represent different strategy/threshold than other centers. Finally, we performed only an internal validation of the REMEMBER score. Prospective studies are needed to externally validate the scoring system before it can be widely applied.

## Conclusions

In our cohort of patients undergoing isolated CABG complicated by PCS requiring VA-ECMO, older age, left main disease, inotropic score > 75, CK-MB > 130 IU/L, serum creatinine > 150 umol/L, and platelet count < 100 × 10^9^/L were identified as pre-ECMO prognosis factors of in-hospital mortality. The REMEMBER score might help clinicians at bedside to predict in-hospital mortality for patients receiving VA-ECMO after CABG for refractory cardiogenic shock.

## Additional file

Additional file 1: **Figure S1.** Flow diagram for selection of patients. **Figure S2.** Number of patients according to the year of extracorporeal membrane oxygenation treatment. **Figure S3.** Study flow chart for all of the included patients. **Figure S4.** Mortality and number of cases according to days on extracorporeal membrane oxygenation. **Figure S5.** (A) Observed mortality in derivation cohort according to pre-ECMO REMEMBER score quartiles (B) Predicted mortality in derivation cohort according to pre-ECMO REMEMBER score quartiles. *N* = number of patients in the study who had particular REMEMBER score. **Figure S6.** The areas under the receiver operating characteristic curves for predicting in-hospital death between early period (2004–2012) and late period (2013–2017). **Table S1.** Outcomes according to hospital survival status. **Table S2.** Pre-ECMO candidate variables associated with hospital mortality by univariate analysis. **Table S3.** Clinical characteristics of the patients at ECMO initiation. **Table S4.** Transformation of continuous variables into categorical variables. **Table S5.** Results of bootstrapping. **Table S6.** Spearman rank correlation matrix for the prediction scoring systems (DOCX 900 kb)

## Abbreviations

AUROC: Area under the receiver operating characteristic curve
CABG: Coronary artery bypass grafting
CCS: Canadian Cardiovascular Society
CK-MB: Creatine kinase-MB
CPB: Cardiopulmonary bypass
CPR: Cardiopulmonary resuscitation
CRRT: Continuous renal replacement therapy
ENCOURAGE: prEdictioN of Cardiogenic shock OUtcome foR Acute myocardial infarction patients salvaGed by VA-ECMO score
IABP: Intra-aortic balloon pump
ICU: Intensive care unit
IQR: Interquartile range
LIMA: Left internal mammary artery
PCS: Postcardiotomy cardiogenic shock
REMEMBER: pRedicting mortality in patients undergoing veno-arterial Extracorporeal MEMBrane oxygenation after coronary artEry bypass gRafting
SAVE: The survival after VA-ECMO score
SOFA: The Sepsis-related Organ Failure Assessment score
VA-ECMO: Venoarterial extracorporeal membrane oxygenation
