# Supplementary material for: Predicting mortality in patients undergoing VA-ECMO after coronary artery bypass grafting: the REMEMBER score
Source: Crit Care. 2019 Jan 11;23:11. doi: 10.1186/s13054-019-2307-y (PMC6330483; doi:10.1186/s13054-019-2307-y)
Supplement: Supplementary file 1 — Figure S1. Flow diagram for selection of patients. Figure S2. Number of patients according to the year of extracorporeal membrane oxygenation treatment. Figure S3. Study flow chart for all of the included patients. Figure S4. Mortality and number of cases according to days on extracorporeal membrane oxygenation. Figure S5. (A) Observed mortality in derivation cohort according to pre-ECMO REMEMBER score quartiles (B) Predicted mortality in derivation cohort according to pre-ECMO REMEMBER score quartiles. N = number of patients in the study who had particular REMEMBER score. Figure S6. The areas under the receiver operating characteristic curves for predicting in-hospital death between early period (2004–2012) and late period (2013–2017). Table S1. Outcomes according to hospital survival status. Table S2. Pre-ECMO candidate variables associated with hospital mortality by univariate analysis. Table S3. Clinical characteristics of the patients at ECMO initiation. Table S4. Transformation of continuous variables into categorical variables. Table S5. Results of bootstrapping. Table S6. Spearman rank correlation matrix for the prediction scoring systems (DOCX 900 kb) [file 13054_2019_2307_MOESM1_ESM.docx]

**Supplemental digital content**

**Predicting mortality in patients undergoing VA-ECMO after coronary artery bypass grafting: the REMEMBER Score**

Liangshan Wang, MD^1^; Feng Yang, MD^1^; Xiaomeng Wang, MD^1^; Haixiu Xie, MD^1^; Eddy Fan, MD, PhD^2^; Mark Ogino, MD^3^; Daniel Brodie, MD, PhD^4^; Hong Wang, MD, PhD^1^; Xiaotong Hou, MD, PhD^1*^

^1^ Center for Cardiac Intensive Care, Capital Medical University Affiliated Anzhen Hospital, Beijing, People’s Republic of China.

^2^ Interdepartmental Division of Critical Care Medicine, University of Toronto, Toronto, Ontario, Canada.

^3^ Division of Neonatology, Nemours/Alfred I. DuPont Hospital for Children, Wilmington, Delaware, USA.

^4^ Columbia University College of Physicians and Surgeons/New York-Presbyterian Hospital, New York, NY, USA.

^*^Corresponding author. Address: Center for Cardiac Intensive Care, Capital Medical University Affiliated Anzhen Hospital, Beijing, People’s Republic of China. Tel: +86-10-64456631; e-mail: [xt.hou@ccmu.edu.cn](mailto:xt.hou@ccmu.edu.cn).

**Methods**

**Weaning criteria**

The patient should have a pulsatile arterial waveform for at least 24 h, be hemodynamically stable, with baseline mean arterial pressure greater than 60 mmHg with no or low doses of catecholamines, with a baseline ECMO flow at 1-1.5 L/min, should have LVEF of ≥ 35%, an aortic VTI of ≥ 12 cm, and a TDSa ≥ 6 cm/s, and have recovered from major metabolic disturbances.

**Data collection**

Two authors (L.W and F.Y) independently reviewed all the medical records in the institutional database and singled out all patients meeting the inclusion criteria. Any discrepancies between the two reviewers were resolved by discussion. Only data from the first ECMO run were analyzed. Baseline characteristics were defined according to the EuroSCORE (1) definition criteria. The following information was collected for each patients: age; sex; weight and body mass index; comorbid conditions; left ventricular ejection fraction; primary diagnosis; EuroSCORE; pre-CABG IABP use; emergency operation; off-pump CABG; left internal mammary artery (LIMA) graft; number of distal anastomoses; transition from cardiopulmonary bypass (CPB) to ECMO ; year of ECMO run; pre-ECMO cardiac arrest; ECMO initiation during cardiopulmonary resuscitation (CPR); CABG-to-ECMO‒implantation time; ECMO inserted in the operating room; pre-ECMO IABP; pre-ECMO continuous renal replacement therapy (CRRT); the Sequential Organ Failure Assessment (SOFA) score (2); the survival after VA-ECMO (SAVE) score (3); the prEdictioN of Cardiogenic shock OUtcome foR Acute myocardial infarction patients salvaGed by VA-ECMO (ENCOURAGE) risk score (4); inotropic score (µg/kg/min, dopamine + dobutamine + 100 × epinephrine + 100 × norepinephrine + 15×milrinone) (5); pre-ECMO hemodynamic parameters; pre-ECMO biological parameters, including PH, HCO_3_^‒^, serum lactate, hemoglobin, hematocrit, platelet, blood urea nitrogen, serum creatinine, and creatine kinase-MB (CK-MB).

**Reference**

1. Nashef SA, Roques F, Michel P, Gauducheau E, Lemeshow S, Salamon R. European system for cardiac operative risk evaluation (EuroSCORE). Eur J Cardiothorac Surg 1999; 16: 9-13.

2. Vincent JL, Moreno R, Takala J, Willatts S, De Mendonça A, Bruining H, et al. The SOFA (Sepsis-related Organ Failure Assessment) score to describe organ dysfunction/failure. On behalf of the Working Group on Sepsis-Related Problems of the European Society of Intensive Care Medicine. Intensive Care Med 1996; 22: 707-10.

3. Schmidt M, Burrell A, Roberts L, Bailey M, Sheldrake J, Rycus PT, et al. Predicting survival after ECMO for refractory cardiogenic shock: the survival after veno-arterial-ECMO (SAVE)-score. Eur Heart J 2015; 36: 2246-56.

4. Muller G, Flecher E, Lebreton G, Luyt CE, Trouillet JL, Bréchot N, et al. The ENCOURAGE mortality risk score and analysis of long-term outcomes after VA-ECMO for acute myocardial infarction with cardiogenic shock. Intensive Care Med 2016; 42: 370-8.

5. Chen YS, Lin JW, Yu HY, Ko WJ, Jerng JS, Chang WT, et al. Cardiopulmonary resuscitation with assisted extracorporeal life-support versus conventional cardiopulmonary resuscitation in adults with in-hospital cardiac arrest: an observational study and propensity analysis. Lancet 2008; 372: 554-61.

**Additional file 1: Figure S1:** **Flow diagram for selection of patients. ECMO, extracorporeal membrane oxygenation; CABG, coronary artery bypass grafting; VV-ECMO, venovenous extracorporeal membrane oxygenation; ECMO-CABG, ECMO initiation before CABG.**

**
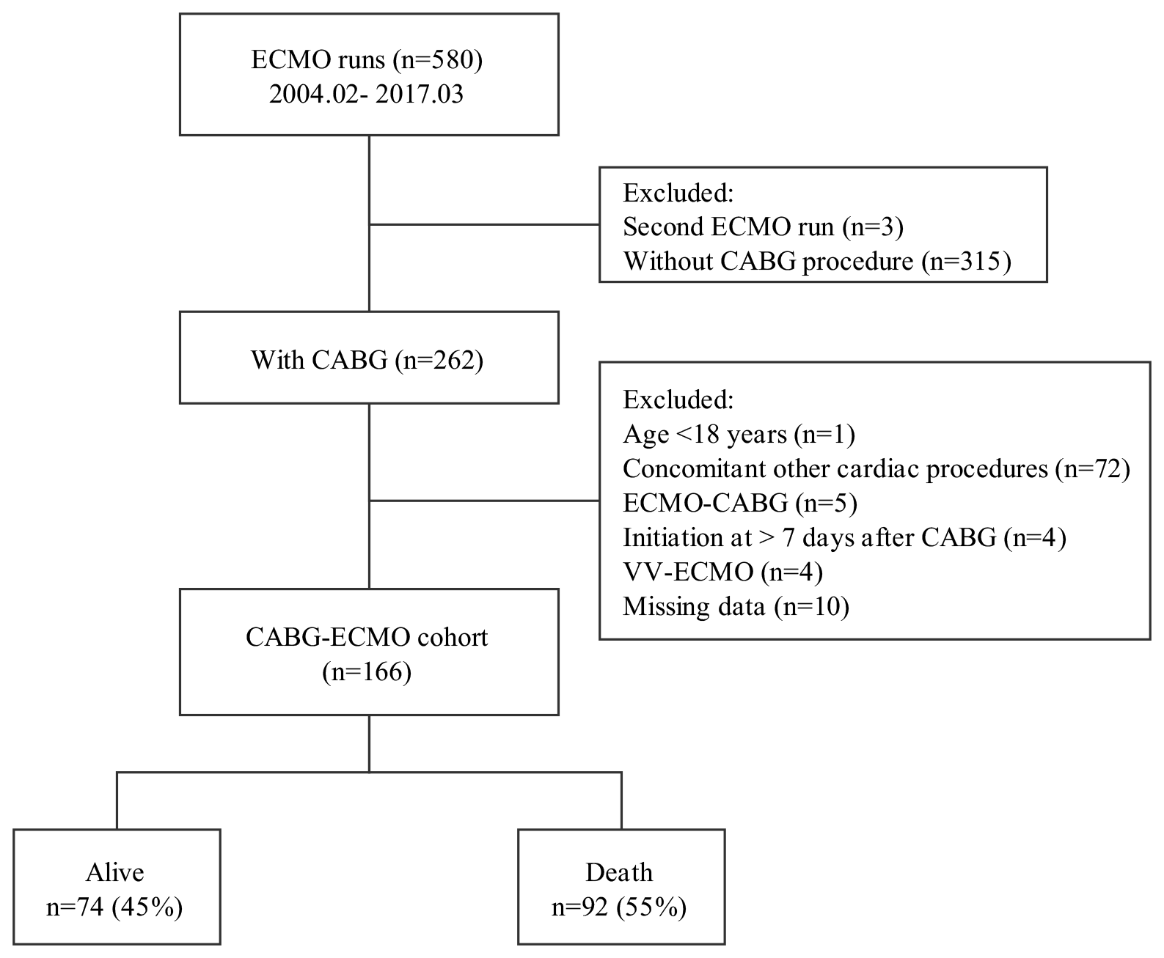
**

**Additional file 1: Figure S2. Number of patients according to the year of extracorporeal membrane oxygenation treatment. ECMO, extracorporeal membrane oxygenation.
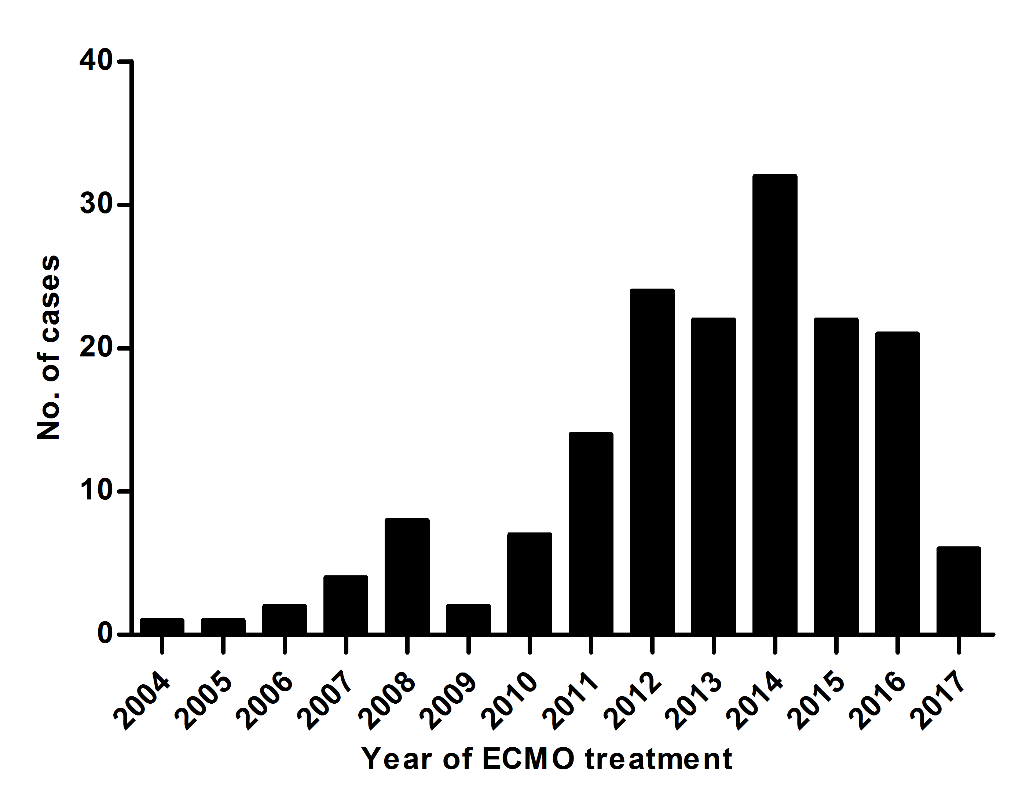
**

**Additional file 1: Figure S3. Study flow chart for all of the included patients. MOF, multiorgan failure. No patients underwent heart transplantation or ventricular assist device.**

**
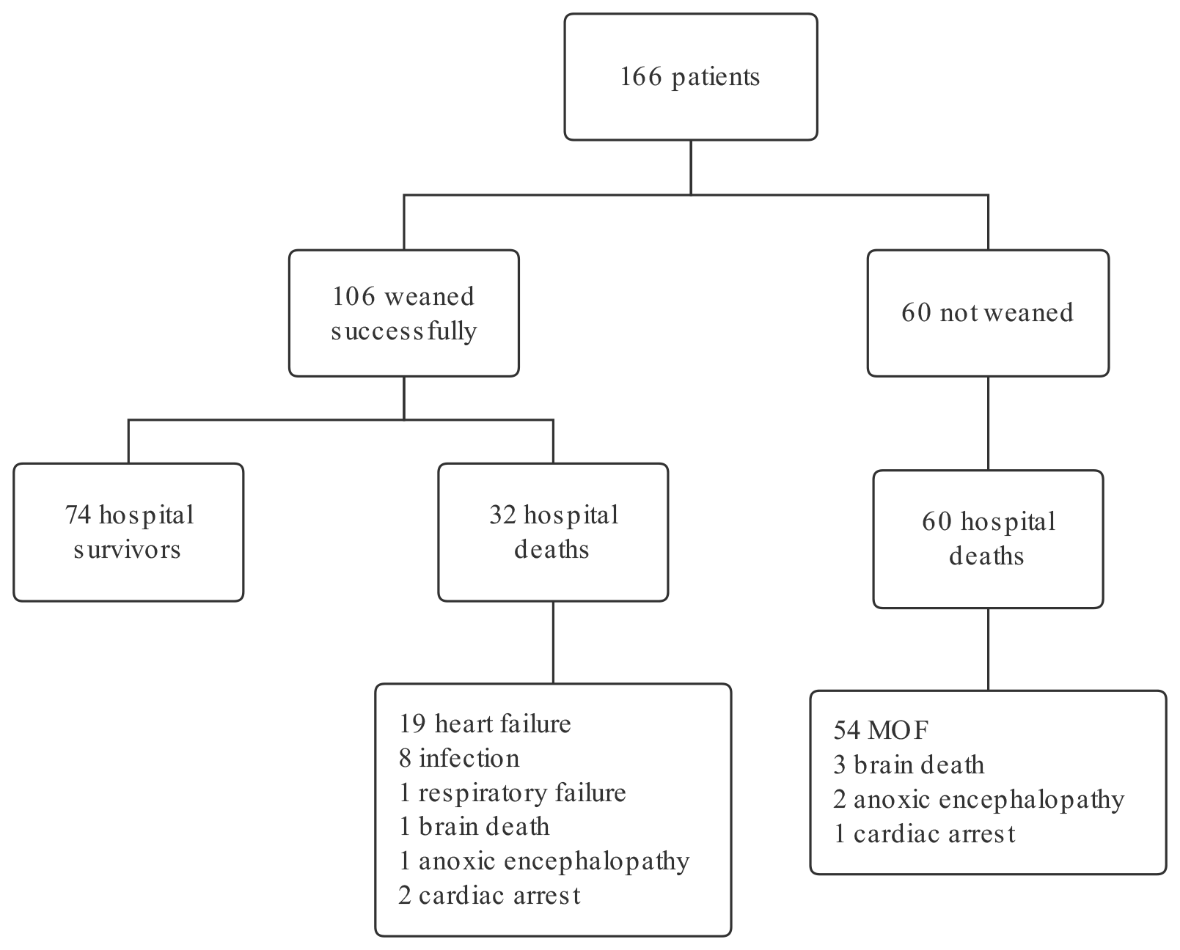
**

**Additional file 1: Figure S4. Mortality and number of cases according to days on extracorporeal membrane oxygenation. Mortality are expressed as mean ± standard Error.**

**

**

**Additional file 1: Figure S5. (A) Observed mortality in derivation cohort according to pre-ECMO REMEMBER score quartiles (B) Predicted mortality in derivation cohort according to pre-ECMO REMEMBER score quartiles. N=number of patients in the study who had particular REMEMBER score.**


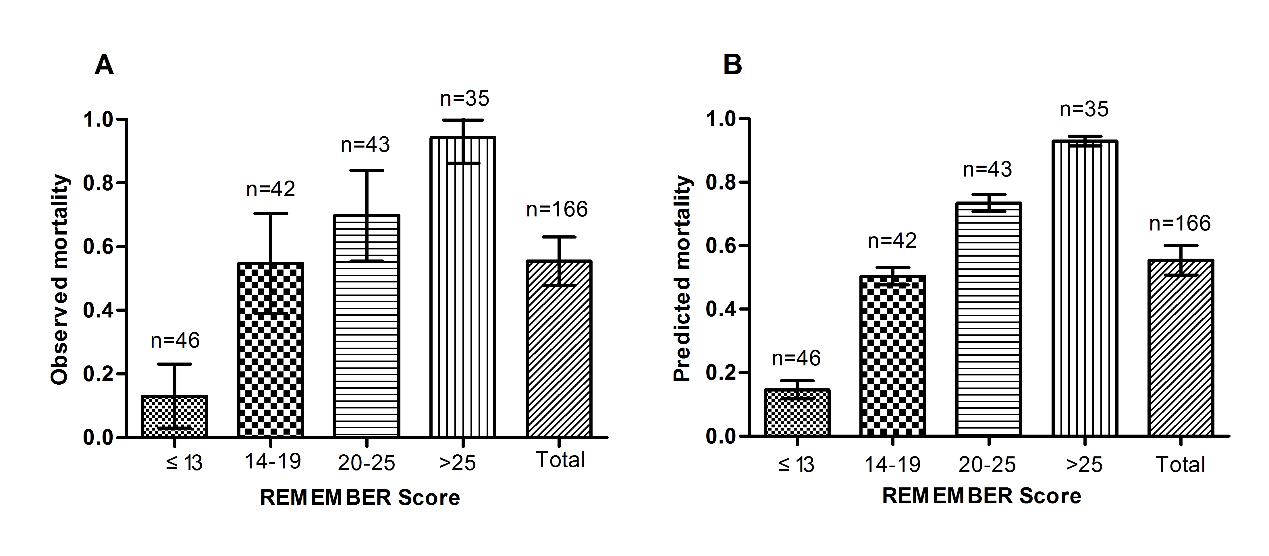


**Additional file 1: Figure S6. The areas under the receiver operating characteristic curves for predicting in-hospital death between early period (2004-2012) and late period (2013-2017).**

**
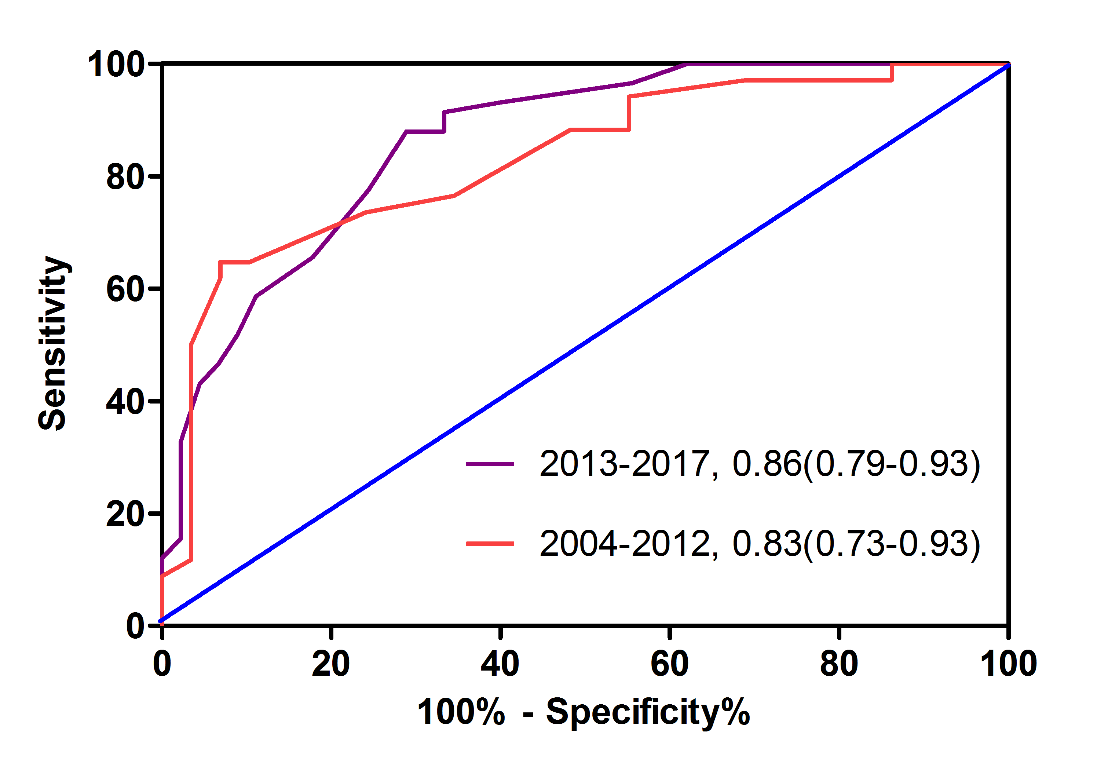
**

**Additional file 1: Table S1. Outcomes according to hospital survival status**

| Outcome variables | All patients (n=166) | Survivors (n=74) | Non-survivors (n=92) | *P* value |
| --- | --- | --- | --- | --- |
| ECMO duration, days | 4 (3-6) | 4 (3-5) | 4 (2-6) | 0.758 |
| Hospital stay, days | 20 (13-30) | 27(20-39) | 16(10-21) | < 0.001 |
| ICU stay, days | 8 (5-12) | 8(7-12) | 7(3-13) | 0.089 |
| Successful weaning off ECMO | 106(64) | 74(100) | 32(35) | < 0.001 |
| CRRT | 72(43) | 20(27) | 52(57) | < 0.001 |
| Systemic infection | 28(17) | 11(15) | 17(18) | 0.537 |
| Bleeding need thoracotomy | 25(15) | 9(12) | 16(17) | 0.349 |
| Gastrointestinal bleeding | 2(1) | 1(1) | 1(1) | 0.877 |
| Major ECMO-related complications | 57(34) | 25(34) | 32(35) | 0.893 |
| Femoral hemorrhage | 19(11) | 5(7) | 14(15) | 0.089 |
| Limb ischemia required  surgical intervention | 15(9) | 6(8) | 9(10) | 0.708 |
| Limb amputation | 2(1) | 0(0) | 2(1) | 0.503 |
| Femoral site infection | 21(13) | 15(20) | 6(7) | 0.008 |
| Cannula thrombosis | 11(7) | 7(9) | 4(4) | 0.316 |
| Change of oxygenator | 10(6) | 6(8) | 4(4) | 0.505 |
| Major neurological complications | 26(16) | 8(11) | 18(20) | 0.123 |
| Brain death | 4(2) | 0(0) | 4(4) | 0.191 |
| Ischemic stroke | 10(6) | 6(8) | 4(4) | 0.494 |
| Hemorrhagic Stroke | 6(4) | 2(3) | 4(4) | 0.884 |
| Anoxic encephalopathy | 6(4) | 0(0) | 6(8) | 0.069 |

Data are presented as medians (25th–75th percentile) or n (%).

ECMO, extracorporeal membrane oxygenation; ICU, intensive care unit; CRRT, continuous renal replacement therapy.

**Additional file 1: Table S2. Pre-ECMO candidate variables associated with hospital mortality by univariate analysis**

| Pre-ECMO variables | OR (95%) | *P* value |
| --- | --- | --- |
| Female | 2.26(1.00-5.09) | 0.049 |
| Age, years |  |  |
| <54 | 1 |  |
| 54-67 | 4.50(1.97-10.27) | <0.001 |
| >67 | 12.5(3.99-39.20) | <0.001 |
| Weight ≥ 83 kg | 0.35(0.14-0.86) | 0.022 |
| Comorbid conditions |  |  |
| Hypertension | 1.86(1.00-3.49) | 0.052 |
| Diabetes | 2.13(1.10-4.14) | 0.026 |
| CCS class 4 angina | 1.66(0.85-3.22) | 0.135 |
| Smoking | 0.55(0.29-1.03) | 0.061 |
| Left main disease^a^ | 3.45(1.67-7.15) | 0.001 |
| Pre-CABG IABP | 2.01(0.89-4.57) | 0.095 |
| Emergency operation | 1.87(0.76-4.61) | 0.174 |
| Inotropic Score >75^b^ | 3.24(1.71-6.16) | < 0.001 |
| DAP ≤ 45 mmHg^b^ | 1.72(0.88-3.40) | 0.115 |
| Pre-ECMO lactate > 9 mmol/L^b^ | 3.38(1.74-6.59) | <0.001 |
| Hemoglobin < 10 g/dL^b^ | 1.72(0.92-3.20) | 0.087 |
| Platelet count< 100×10^9^/L^b^ | 2.14(1.14-3.99) | 0.017 |
| Serum creatinine > 150 umol/L^b^ | 5.56(2.47-12.49) | <0.001 |
| CK-MB >130 IU/L^b^ | 2.14(1.15-4.00) | 0.017 |

OR, odds ratio; ECMO, extracorporeal membrane oxygenation; CCS, Canadian Cardiovascular Society classification of angina; CABG, coronary artery bypass grafting; IABP, intra-aortic balloon pump; DAP, diastolic arterial blood pressure; CK-MB, creatine kinase-MB.

^a^ Left main disease was defined as any stenosis ≥50 % of the left main trunk.

^b^ Worse value within 6 h prior ECMO cannulation.

**Additional file 1: Table S3. Patients’ characteristics at ECMO initiation**

| Characteristic | All patients (n=166) | Survivors (n=74) | Non-survivors (n=92) | *P* value |
| --- | --- | --- | --- | --- |
| BMI, kg/m^2^ | 25(23-27) | 25(23-27) | 25(23-27) | 0.447 |
| Comorbid conditions |  |  |  |  |
| Dyslipidemia | 52(31) | 21(28) | 31(34) | 0.463 |
| Stroke | 15(9) | 5(8) | 10(11) | 0.358 |
| Recent MI | 44(27) | 18(24) | 26(28) | 0.568 |
| Chronic pulmonary disease | 5(3) | 3(4) | 2(2) | 0.481 |
| Peripheral artery disease | 19(11) | 8(11) | 11(12) | 0.818 |
| Previous PCI | 30(18) | 13(18) | 17(18) | 0.880 |
| Previous CABG | 3(2) | 1(1) | 2(2) | 0.693 |
| Pre-CABG LVEF, % | 57(48-64) | 56(49-65) | 58(45-64) | 0.565 |
| Year of ECMO |  |  |  | 0.698 |
| 2004-2008 | 16(10) | 6(8) | 10(11) |  |
| 2009-2012 | 47(28) | 23(31) | 24(26) |  |
| 2013-2017 | 103(62) | 45(61) | 58(63) |  |
| CABG to ECMO < 24h | 136(82) | 63(85) | 73(79) | 0.335 |
| ECMO inserted in OR | 98(59) | 43(58) | 55(60) | 0.827 |
| Pre-ECMO CRRT | 7(4) | 2(3) | 5(5) | 0.630 |
| SAVE score | -2(-5 to 0) | -1(-4 to 2) | -4(-6 to -1) | <0.001 |
| ENCOURAGE score | 22（16-27） | 17（14-22） | 24（17-28） | <0.001 |
| Hemodynamic paramaters ^a^ |  |  |  |  |
| Pulse pressure, mmHg | 25(20-30) | 25(20-30) | 25(20-30) | 0.793 |
| Heart rate, /min | 118(106-130) | 117(106-131) | 118(105-130) | 0.956 |
| Respiratory rate, /min | 14(12-15) | 13(12-14) | 14(12-16) | 0.215 |
| Peak inspiratory pressure, cmH_2_O | 18(16-22) | 18(16-22) | 18(16-22) | 0.907 |
| Biological parameters^a^ |  |  |  |  |
| HCO_3_^‒^, mmol/L | 22(19-25) | 23(19-25) | 22(18-25) | 0.217 |
| Hematocrit, % | 29(26-32) | 30(27-32) | 28(25-32) | 0.085 |
| BUN^b^, mmol/L | 6.1(5.0-8.2) | 5.7(4.6-6.8) | 6.8(5.0-8.6) | 0.112 |

Data are presented as medians (25th–75th percentile) or n (%).

ECMO, extracorporeal membrane oxygenation; BMI, body mass index; MI, myocardial infarction; PCI, percutaneous coronary intervention; CABG, coronary artery bypass grafting; LVEF, left ventricular ejection fraction; OR, operation room; CRRT, continuous renal replacement therapy; BUN, blood urea nitrogen.

EuroSCORE was collinear to age; HCO3^‒^ and PH were collinear to lactate; hematocrit was collinear to hemoglobin; blood urea nitrogen was collinear to creatinine. EuroSCORE, HCO3^‒^, PH, hematocrit and blood urea nitrogen were not included in our model.

^a^ Worse value within 6 h prior ECMO cannulation.

^b^ Values were obtained for 163/166 patients (73 survivors and 90 on-survivors).

**Additional file 1: Table S4. Transformation of continuous variables into categorical variables**

| Variable |  |  | Quartile (mortality) |  |
| --- | --- | --- | --- | --- |
| (25^th^, 50^th^,75^th^ percentiles) | 1 | 2 | 3 | 4 |
| Age (54, 61, 67) | 28 | 59 | 61 | 81 |
| Weight (63, 70, 80) | 57 | 58 | 50 | 54 |
| EuroSCORE (4,6,7) | 42 | 48 | 33 | 75 |
| Inotropic score (55, 75, 93) | 30 | 53 | 69 | 71 |
| DAP (30,40,50) | 57 | 59 | 57 | 43 |
| Lactate (7.8, 11.4, 17.4) | 34 | 53 | 53 | 81 |
| Hemoglobin (8.6, 9.8, 10.7) | 67 | 60 | 40 | 51 |
| Platelet (58, 103,153) | 63 | 68 | 48 | 43 |
| Creatinine (91, 121, 160) | 19 | 50 | 59 | 85 |
| CK-MB (48, 143, 259) | 45 | 50 | 56 | 71 |

DAP, diastolic arterial blood pressure; CK-MB, creatine kinase-MB.

Median values were chosen for inotropic score, hemoglobin, and platelet. Age was trichotomized because mortality was comparable for quartiles 2 and 3 but markedly different for quartiles 1 and 4. The breakpoints was ~46, ~6, ~83, ~9.3, ~145 and ~126 for DAP, EuroSCORE, weight, lactate, creatinine and CK-MB, respectively. For practical purposes, values were rounded to the nearest integer.

**Additional file 1: Table S5. Results of bootstrapping**

|  |  | Bootstrap | | | |
| --- | --- | --- | --- | --- | --- |
| Parameter | β Coefficient | Bias | SE | *P* value | 95% CI |
| Age, years |  |  |  |  |  |
| 54-67 | 1.783 | 0.105 | 0.599 | 0.001 | 0.747-3.142 |
| >67 | 2.384 | 0.183 | 1.197 | 0.002 | 1.036-4.343 |
| Left main disease | 1.625 | 0.115 | 0.569 | 0.003 | 0.699-2.911 |
| Inotropic score >75 | 1.126 | 0.117 | 0.537 | 0.009 | 0.267-2.380 |
| CK-MB> 130 IU/L | 1.145 | 0.064 | 0.473 | 0.009 | 0.388-2.237 |
| Serum creatinine>150 umol/L | 1.496 | 0.161 | 0.592 | 0.001 | 0.631-3.030 |
| Platelet count < 100 G/L | 1.271 | 0.123 | 0.505 | 0.006 | 0.495-2.516 |
| Constant | -3.885 | -0.283 | 0.870 | 0.001 | -6.162 to -2.780 |

All β Coefficients in bootstrapping attained the levels of highly significance and bias values were very small, confirming the stability of the original model.

**Additional file 1: Table S6. Spearman rank correlation matrix for the prediction scoring systems**

|  | REMEMBER | EuroSCORE | SAVE | ENCOURAGE | SOFA |
| --- | --- | --- | --- | --- | --- |
| REMEMBER | 1.000 |  |  |  |  |
| EuroSCORE | 0.192 | 1.000 |  |  |  |
| SAVE | -0.423 | -0.264 | 1.000 |  |  |
| ENCOURAGE | 0.496 | 0.178 | -0.412 | 1.000 |  |
| SOFA | 0.542 | 0.068 | -0.328 | 0.500 | 1.000 |
